# Analysis of Nuclear Encoded Mitochondrial Gene Networks in Cervical Cancer

**DOI:** 10.31557/APJCP.2021.22.6.1799

**Published:** 2021-06

**Authors:** Cecile Meneur, Sangavi Eswaran, Divya Adiga, Sriharikrishnaa S, Nadeem Khan G, Sandeep Mallya, Sanjiban Chakrabarty, Shama Prasada Kabekkodu

**Affiliations:** 1 *Department of Cell and Molecular Biology, Manipal School of Life Sciences, Manipal Academy of Higher Education, Manipal-576104, Karnataka, India. *; 2 *La Rochelle University, Avenue Albert Einstein, 17031, La Rochelle, France. *; 3 *Department of Bioinformatics, Manipal School of Life Sciences, Manipal Academy of Higher Education, Manipal-576104, Karnataka, India. *

**Keywords:** Nuclear encoded mitochondrial genes, prognosis, survival, TCGA, CESC, cervical cancer

## Abstract

**Background:**

Cervical cancer (CC) is one of the most common female cancers in many developing and underdeveloped countries. High incidence, late presentation, and mortality suggested the need for molecular markers. Mitochondrial defects due to abnormal expression of nuclear-encoded mitochondrial genes (NEMG) have been reported during cancer progression. Nevertheless, the application of NEMG for the prognosis of CC is still elusive. Herein, we aimed to investigate the associations between NEMG and CC prognosis.

**Materials and Methods:**

The differentially expressed genes (DEG) in the TCGA-CESC dataset and NEMGs were retrieved from TACCO and Mitocarta2.0 databases, respectively. The impact of methylation on NEMG expression were predicted using DNMIVD and UALCAN tools. HCMDB tool was used to predict genes having metastatic potential. The prognostic models were constructed using DNMIVD, TACCO, GEPIA2, and SurvExpress. The functional enrichment analysis (FEA) was performed using clusterProfiler. The protein-protein interaction network (PPIN) was constructed to identify the hub genes (HG) using String and CytoHubba tools. Independent validation of the HG was performed using Oncomine and Human Protein Atlas databases. The druggable genes were predicted using DGIdb.

**Results:**

Among the 52 differentially expressed NEMG, 15 were regulated by DNA methylation. The expression level of 16, 10, and 7 has the potential for CC staging, prediction of metastasis, and prognosis. Moreover, 1 driver gene and 16 druggable genes were also identified. The FEA identified the enrichment of cancer-related pathways, including AMPK and carbon metabolism in cancer. The combined expression of 10 HG has been shown to affect patient survival.

**Conclusion:**

Our findings suggest that the abnormal expression of NEMGs may play a critical role in CC development and progression. The genes identified in our study may serve as a prognostic indicator and therapeutic target in CC.

## Introduction

Cervical cancer (CC) is a significant public health problem affecting women in countries with low resource settings. According to the Global Cancer Observatory database, there were 570,000 cases and 311,000 deaths due to CC in 2018 (Ferlay et al., 2018). Over a third of the overall global CC cases were contributed together by India and China. In 2018, the CC cases in India and China were 97,000 and 106,000, respectively, with mortality of 60,000 and 48,000, respectively (Arbyn et al., 2020; Bray et al., 2018). The Squamous cell carcinoma (SCC) is the most common CC histological type. The standard method for CC treatment includes surgery, chemotherapy, and radiotherapy. Persistent infection with high-risk HPV, high parity, multiple sexual partners, smoking cigarettes, and long-term use of oral contraceptive pills are a few risk factors associated with CC. The introduction of Pap testing and HPV typing has significantly reduced CC incidence in many countries (Johnson et al., 2019; Wudtisan et al., 2019). Despite the availability of effective early screening and detection methods, CC is generally diagnosed at an advanced stage leading to a high mortality rate. The 5-year survival rate for stage 1, stage II, stage III, and stage IV CC was 81-96%, 65-87%, 35-50%, and 15-20%, respectively. CC with advanced stage shows recurrence and therapy resistance and most patients succumb within three years (Canfell et al., 2020; Charakorn et al., 2018). This suggests the need for biomarkers to identify the patients with poor prognosis at an early stage for intensified treatment for improved patient care. In this direction, identifying the molecular markers and associated networks may be useful for better management of CC.

Genome-wide genetic and epigenetic studies using normal and tumor samples lead to identifying cancer-associated genes and related pathways for diagnostic and prognostic applications in cancer. Studies have shown that the genome-wide data’s reanalysis may provide (i) molecular markers for diagnosis, prognosis, therapy, and (ii) a novel gene-gene regulatory networks, mechanisms, and pathways facilitating CC. Interestingly, previous studies have indicated that the integrated bioinformatics analysis of big data generated from cancer studies can develop reliable and novel biomarkers, networks, and mechanisms related to CC pathogenesis (Lin et al., 2019; Wu et al., 2019).

Mitochondria are an intracellular organelle that plays a role in cellular bioenergetics, free radical generation, and cell signaling. The human mtDNA is 16.5kb in size and codes for 37 genes linked with the OXPHOS pathway, ATP production, and apoptosis initiation (Shaughnessy et al., 2015). Defects in both structure and function of mitochondria contribute to resistance to apoptosis, abnormal cell proliferation, and therapy resistance in cancer (Indran et al., 2011). Both somatic mutation and mtDNA copy number changes are linked with cancer, including CC (Chatterjee et al., 2006; Sun et al., 2020). Although mtDNA encodes for proteins, most of the protein required for its function is synthesized by nuclear-encoded genes. As per the estimate, over 1,500 proteins are necessary for maintaining the structure and function of mitochondria. The anterograde and retrograde signaling between mitochondria and the nucleus is critical for the normal functioning of the cell (Pfanner et al., 2019). Many nuclear-encoded genes linked with mitochondrial function (NEMG) show a significant difference in their expression in normal and CC conditions (Sun et al., 2020). Therefore, profiling the expression of NEMG can be useful as a sensitive and specific biomarker for diagnostic and prognostic applications in CC.

In the present study, we have investigated the differentially expressed nuclear-encoded mitochondrial genes (DE-NEMG) and the associated network for diagnostic and prognostic applications in CC. We have analyzed The Cancer Genome Atlas, Cervical Squamous Cell Carcinoma and Endocervical Adenocarcinoma (TCGA-CESC) datasets for DE-NEMG in CC to evaluate its prognostic significance. Using the TCGA-CESC dataset, we have constructed the protein-protein interaction network (PPIN) and hub genes (HG) and predicted the possible drug targets. The integrated bioinformatic analysis identified interaction networks and mitochondrial targets that may be useful as a potential marker for diagnosis, treatment, and CC therapy.

## Materials and Methods

*Selection of NEMG*

MitoCarta2.0 contains human and mouse proteins having strong mitochondrial translocation signals from fourteen different tissue samples (Calvo et al., 2016). In the present study, we downloaded 1158 NEMGs from the MitoCarta2.0 database to identify the DE-NEMGs. 

*TCGA-CESC datasets*

The TCGA contains molecularly characterized epi (genomics), transcriptomics, and proteomics data for over 20000 primary tumors and matched normal samples across 33 different cancer types. The TCGA-CESC dataset contains clinical and molecular data for 310 samples (307 tumors and 3 normal samples) (Tomczak et al., 2015). The differentially expressed genes in TCGA-CESE data sets were retrieved using the Transcriptome Alterations in Cancer Omnibus (TACCO: http://tacco.life.nctu.edu.tw/) online tool. The TACCO is a freely available online tool for identifying differentially expressed genes (DEG) and miRNAs from TCGA datasets. The TACOO can be used for Gene Ontology (GO), pathway enrichment, and prognostic model construction using a user-defined gene list (Chou et al., 2019).

*Identification of DE-NEMG*

We have downloaded a list of 1,158 NEMGs from the MitoCarta2.0 database. We downloaded the list of differentially expressed protein-coding genes (DEGs) between normal and tumor samples in the TCGA-CESC dataset via the TACCO online tool. We have identified the DEGs in the TCGA-CESC data set using the “select DEGs” function with cut-off criteria of adjusted p-value < 0.05 and expression value log 2-fold change of +2 and -2 between tumor and normal tissue samples. We have compared the DEGs in TCGA-CESC dataset with that of NEMG downloaded from the MitoCarta2.0 using Venny 2.1 (https://bioinfogp.cnb.csis.es/tools/venny/) online tool to identify the DE-NEMGs.

*Identification of differentially methylated NEMG*

We have used UALCAN (http://ualcan.path.uab.edu/index.html) and DNMIVD (http://119.3.41.228/dnmivd/index/) online resources for identification of methylation regulated NEMGs. UALCAN online tool can identify differentially expressed, methylation-regulated genes, along with clinical attributes such as survival, age, histology, tumor grade, and nodal metastasis. A beta values of 0 and 1 are considered as unmethylated and completely methylated respectively. Beta value: 0.7 - 0.5 and 0.3 -0.25 and a P<0.05 considered significantly hypermethylated and hypomethylated (Chandrashekar et al., 2017). DNMIVD is a user-friendly interactive visualization of the DNA methylation profile of genes in TCGA datasets. The tool has a module for gene expression analysis, methylation and expression correlation, survival analysis, diagnostic and prognostic model generation. The samples were divided into high (H) and low (L) based on median DNA methylation beta values as a threshold. DNA methylation beta values of 0.3 and 0.7 were used as a cutoff for sample categorization (Ding et al., 2020).

*Evaluation of metastatic potential of DE-NEMG*

The Human Cancer Metastasis Database (HCMDB https://hcmdb.i-sanger.com/) is a freely available online tool for assessing gene metastatic potential. The interactive web tool contains metastatic data from 38 metastasis sites and 29 cancer types (Zheng et al., 2018). In the present study, the HCMDB database was used to test the metastatic potential of DE-NEMG in TCGA-CESC data. 

*Construction of PPIN and identification HG*

The PPIN and HG play a crucial role in governing the biological process and signaling pathways. They are commonly used to predict cellular function, understand disease mechanisms, and design drug targets. Using the Search Tool for the Retrieval of Interacting Genes (STRING: https://string-db.org/) version 11, the PPIN of the DE-NEMG was constructed with the highest confidence score of 0.9 and minimum interaction number =2 (Szklarczyk et al., 2017). The cytoHubba V 0.1 plugin of Cytoscape was used to identify the HG of the PPIN. The 10 HG were selected based on the highest degree of connectivity on the PPIN. The two-dimensional visualization of the network was performed through Cytoscape 3.7.2 (https://cytoscape.org/ (Chin et al., 2014; Shannon et al., 1971).

*Functional enrichment analysis (FEA) of DE-NEMG*

The FEA included prediction of gene ontology (GO) [biological process (BP), cellular component (CComp) and molecular function (MF)], and pathway enrichment analysis using the Kyoto Encyclopedia of Genes and Genomes (KEGG: http://www.genome.jp) (Kanehisa, 2000). The analysis and visualization of functional and pathway enrichment for 52 DE-NEMG were performed using the clusterProfiler tool in the Bioconductor package in the R statistical environment (Yu et al., 2012).

*Survival analysis of DE-NEMG*

In the present study, prognostic prediction and pathological classification models were generated for CC using the 52 differentially expressed NEMG via the Random forest algorithm using TACCO online tool. Using GEPIA2 (http://gepia2.cancer-pku.cn/#index), we have predicted the association between DE-NEMGs with disease-free survival (DFS) and or overall survival (OS) at the individual gene level (Tang et al., 2019).

*Identification of driver gene and enrichment analysis*

The driver gene analysis was carried out using DriverDBv3 (http://driverdb.tms.cmu.edu.tw/) online tool. The DriverDBv3 uses genetic and epigenetic changes in OMICS databases to compare against clinical data to identify cancer driver genes (Liu et al., 2020). We have used the CCDB database (https://webs.iiitd.edu.in/raghava/ccdb/index.php) (Agarwal et al., 2011) to identify the DE-NEMG, which are already reported in CC. Also, the disease enrichment analysis of 52 DE-NEMG was performed using Comparative Toxicogenomics Database (http://ctdbase.org) (Davis et al., 2019).

*Analysis of the HG*

We evaluated the relative expression 10 HG via the GEPIA2 tool. The GEPIA2 is an online tool for in silico differential expression analysis of genes using TCGA transcriptome and GTEx expression data. It allows the expression analysis between normal and tumor samples, multiple gene comparisons, and survival analysis. We analyzed the survival data of 10 HGs using TCGA-CESC data. Next, we analyzed the expression of 10 HGs at the protein level using The Human Protein Atlas (HPA: http://www.proteinatlas.org/) (Thul and Lindskog, 2018).

*Drug and DE-NEMG interaction analysis*

The drug-gene interaction database (DGIdb, https://www.dgidb.org/) was used to screen the drugs that interact with DE-NEMGs. The DGIdb is a user-friendly online resource for screening a druggable genome. The tool has browse and search options to identify DGI and enrichment. Besides, the DGIdb has options for searching approved, antineoplastic, and immunotherapies options to filter the drugs (Cotto et al., 2018).

## Results

*Data Retrieval and differential gene expression analysis*

**Figure 1:**
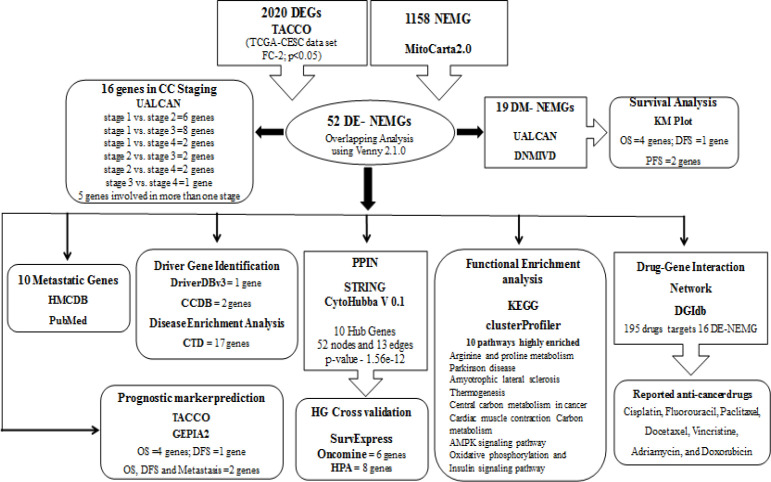
The Flow Diagram of Bioinformatic Analysis NEMG and Associated Pathways in CC

**Figure 2:**
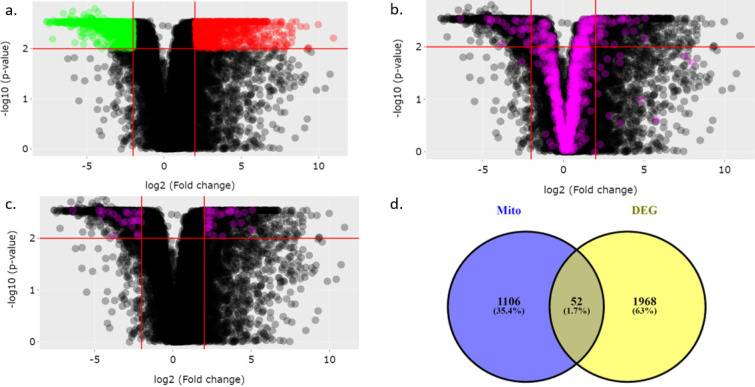
Nuclear Encoded Mitochondrial Genes (NEMG) are Differentially Expressed between Normal and Cervical Cancer Samples. A) Represents the expression profile of genes in TCGA-CESC dataset. B) Represents the expression profile of 1158 NEMG in TCGA-CESC dataset. C) Represents the expression profile of 52 DE-NEMGs in TCGA-CESC dataset. D) Venn diagram showing 52 DE-NEMGs

Figure 1 depicts the workflow of the study. We first retrieved the DEGs in the TCGA-CESC dataset using the TACCO database. Our analysis identified 2020 (Figure 2A) DEGs with a fold change of 2 and P<0.05. Among those 2020 DEGs, 802 and 1218 genes were upregulated and downregulated, respectively. The MitoCarta2.0 database contained 1158 NEMG. Figure 2B shows the expression of 1158 NEMG in the TCGA-CESC dataset. The comparison between 2020 DEGs with that of 1158 NEMG lead to the identification of 52 (27 upregulated and 25 downregulated) DE- NEMG (Table 1, Figure 2C, and 2D). The list of all DEGs in the TCGA-CESC dataset and NEMG retrieved from Mitocarta2.0 are shown in Supplementary Tables 1, 2 and 3.

*DNA methylation regulated NEMG*

Next, we analyzed the impact of promoter DNA methylation on the expression of 52 DE-NEMG using DNMIVD tools by Pearson’s correlation analysis. We considered genes that are commonly predicted as differentially methylated by the UALCAN and DNMIVD tools for all subsequent analysis. Among the 52 differentially expressed genes, 19 genes showed significant aberrant promoter methylation. Among the 19 differentially methylated genes, 8 and 11 genes were hypomethylated and hypermethylated, respectively (Table 1). Furthermore, 27 genes showed an inverse correlation between methylation and expression by Pearson correlation analysis (Table 1, Supplementary Figure 1). Out of 27 genes, 19 of them showed significant differential methylation between normal and tumor samples. Interestingly, among the 19 significantly differentially methylated genes, only 15 revealed the inverse correlation between methylation and expression (Supplementary Figure 2).

*Identification of DE-NEMG for CC staging application*

By using UALCAN, we analyzed the expression of 52 DE-NEMG in various stages of CC. Among the DEGs, 16 of them showed the potential to differentiate CC stages (Table 1). Expression analysis of 6 genes (PIF1, DHCR24, CPT1B, FASN, DMGDH, and PDK4) can distinguish stage 1 from stage 2 CC. Genes, namely, HK2, NCEH1, NIPSNAP3B, ABCD2, ACACB, DEPP1, ACSS3, and PABPC5, were differentially expressed between stage 1 vs. stage 3 CC. NIPSNAP3B and DMGDH expression were significantly different in stage 1 vs. stage 4 CC. Gene, namely, NCEH1 and ABCD2 expression, was significantly different in stage 2 vs. stage 3 CC. SLC25A10 and NIPSNAP3B expression showed significant differential expression between stage 2 vs. stage 4 CC. ACACB expression was significantly different between stage 3 vs. stage 4 CC. Interestingly, genes such as NCEH1, NIPSNAP3B, ABCD2, DMGDH, and ACACB showed a significant difference in expression in more than one CC stage (Supplementary Table 4). 

*Identification of NEMG for prognostic application*

**Figure 3:**
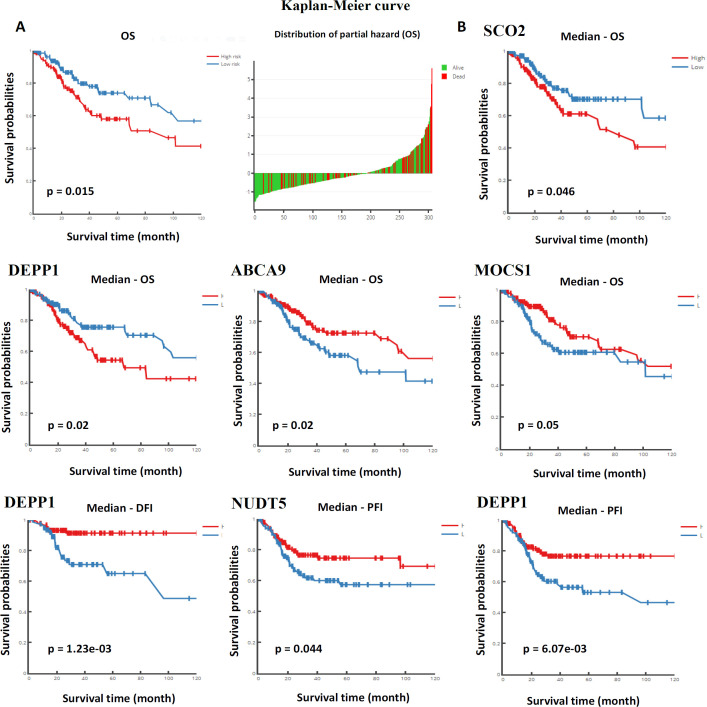
Prognostic Model Based on DNA Methylation. A) Represents the KM plot generated using multivariate proportional hazard regression model based on the 19 differentially methylated genes. B) Represents the KM plot for differentially methylated individual genes linked with OS, DFS and PFS

We investigated the differentially methylated and DEGs for their predictive utility in CC using DNMIVD, UALCAN, and TACCO tools with default parameters. The multivariate proportional hazard regression model was applied to divide the patients into high-risk and low-risk groups to generate the Kaplan Meier (KM) plot. The prognostic model generated using 19 differentially methylated NEMG suggested a significant prognostic value in CESC (Figure 3A). The high-risk category patients showed lower OS as opposed to low-risk categories. When analyzed individually, the differential methylation of gene promoters had substantial implications in CC prognosis (Figure 3B). Methylation status of SCO2, MOCS1, DEPP1, and ABCA9 was linked with OS, and DEPP1 was linked with DFS, and NUDT5, DEPP1 with PFS (Figure 3B) (Table 1).

**Figure 4:**
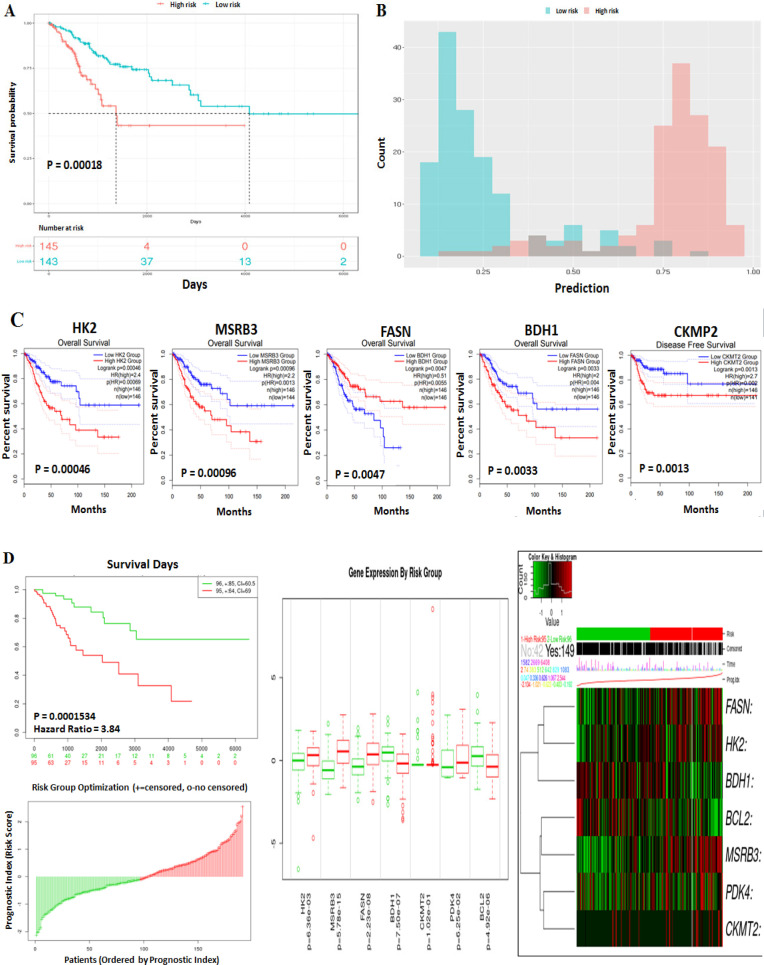
Survival Analysis of DE-NEMGs. A) The association between DE-NEMG with CC prognosis generated using random forest model. B) Represents the sensitivity and specificity of DE-NEMG. C) Represents the KM plot of DE-NEMG associated with OS and DFS. D) Represents the KM plot and risk score construction using 52 DE-NEMGs

Using the Random Forest model, we assessed the association between DE-NEMG with CC prognosis using the TACCO tool (Figure 4A). The DE-NEMGs displayed a sensitivity and specificity of 0.91 and 0.89, respectively, to distinguish high risk from low-risk categories (Figure 4B). The prognostic ability of the individual genes was tested by the GEPIA2 tool using Cox Proportional-Hazards Model. The genes with significant OS (499 genes) and DFS (500 genes) in the TCGA-CESC dataset were downloaded from GEPIA2 and compared against 52 DE-NEMG using Venny 2.1.0 tool. We found that genes, namely, HK2, MSRB3, FASN, and BDH1, showed a significant prognostic value towards OS; while CKMT2 towards DFS (Figure 4C) (Table 1). Besides, PDK4 and BCL2 are additional genes significantly associated with (i) OS and DFS and (ii) metastasis by literature analysis. Next, these genes (HK2, MSRB3, FASN, BDH1, CKMT2, PDK4, and BCL2) were used for risk score construction using the SurvExpress tool (http://bioinformatica.mty.itesm.mx:8080/Biomatec/SurvivaX.jsp) (Aguirre-Gamboa et al., 2013). CC patients were categorized into high-risk and low-risk groups as per the median risk score for survival outcomes. The KM plot suggested that the high-risk patients showed a significantly lower survival rate than the low risk-group (Figure 4D).

*Identification of DE-NEMG associated with metastasis*

The DE-NEMG with metastatic potential in CC was identified using HCMDB online tool and by literature search using PubMed. Among the 52 DEGs, 10 genes (BDH1, CPT1B, CKMT1A, MSRB3, MGARP, PMAIP1, TDRKH, CKMT1B, PDK4, and BCL2) were linked with head and neck, lung, or lymph node metastasis (Table 1). 

*PPIN and HG analysis*

**Figure 5:**
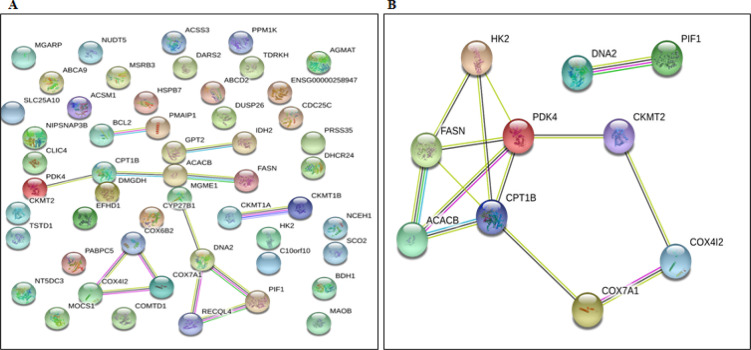
PPIN of DE-NEMG. A) Represents the PPIN of 52 DE-NEMGs. B) Represents the PPIN of 19 hub genes

The PPINs of 52 DEGs were generated using the STRING database. The PPIN of DE-NEMG displayed 52 nodes and 13 edges with a PPIN enrichment p-value of 1.56e-12 (Figure 5A). The top 10 connection proteins or HG were predicted using the CytoHubba tool and visualized via the Cytoscape tool. The CytoHubba analysis identified FASN, HK2, ACACB, PIF1, COX7A1, CKMT2, CPT1B, PDK4, DNA2, and COX4I2 as top 10 Hub genes, and these 10 proteins may play a key role in CC development or progression (Figure 5B). 

*Driver Gene Identification*

The DriverDBv3 database has predicted 337 genes as a potential driver gene in the TCGA-CESC dataset. The comparison of 52 DE-NEMG with that of 337 identified IDH2 as a potential driver gene. In addition, the CCDB database compared 538 genes reported in CC with 52 DE-NEMG identified 2 common genes (BCL2, DUSP26). The disease enrichment analysis of 52 DE-NEMG using Comparative Toxicogenomics Database identified 17 genes (ABCA9, ABCD2, ACSM1, BCL2, CDC25C, CLIC4, CPT1B, DEPP1, EFHD1, FASN, HK2, IDH2, MAOB, PDK4, PMAIP1, SLC25A10, and TUBB3) associated with various cancers.

*Functional enrichment analysis of DE-NEMG*

**Figure 6:**
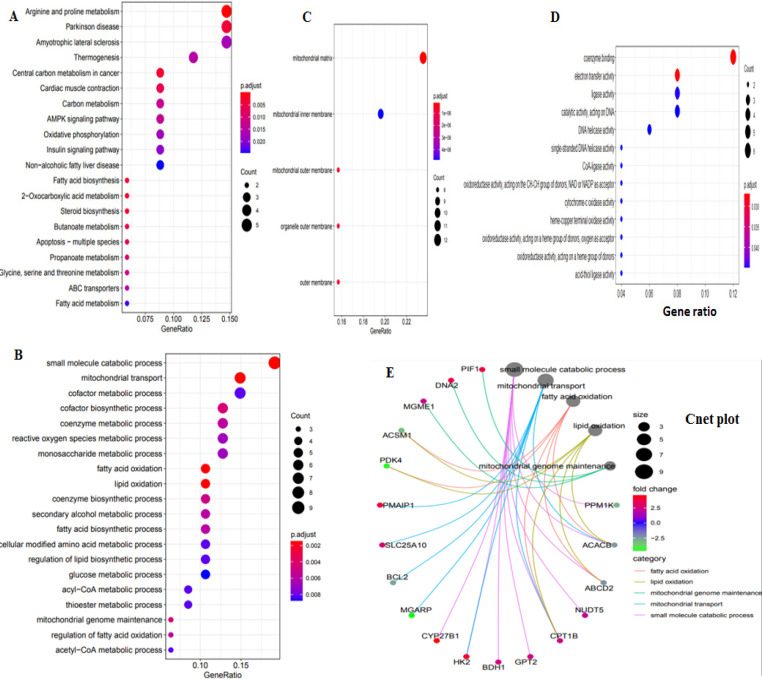
Functional and Pathway Enrichment Analysis of DE-NEMG. A) Signaling pathways analysis (B) biological process (C) cellular components (D) molecular components. E) Cent plot represent connection between genes and enriched ontology terms

The GO and KEGG pathway analysis were carried out for 52 DE-NEMG using clusterProfiler to understand their function in CC (Figure 6A). The pathway enrichment using KEGG annotations identified genes related to pathways such as arginine and proline metabolism, Parkinson disease, Amyotrophic lateral sclerosis, Thermogenesis, Central carbon metabolism in cancer, Cardiac muscle contraction, Carbon metabolism, AMPK signaling pathway, Oxidative phosphorylation, and Insulin signaling pathway as highly enriched. The 52 differentially expressed NEMG linked with biological process, cellular components, and molecular functions are shown in Figure 6B-6E.

*HG cross validation in independent datasets*

**Figure 7:**
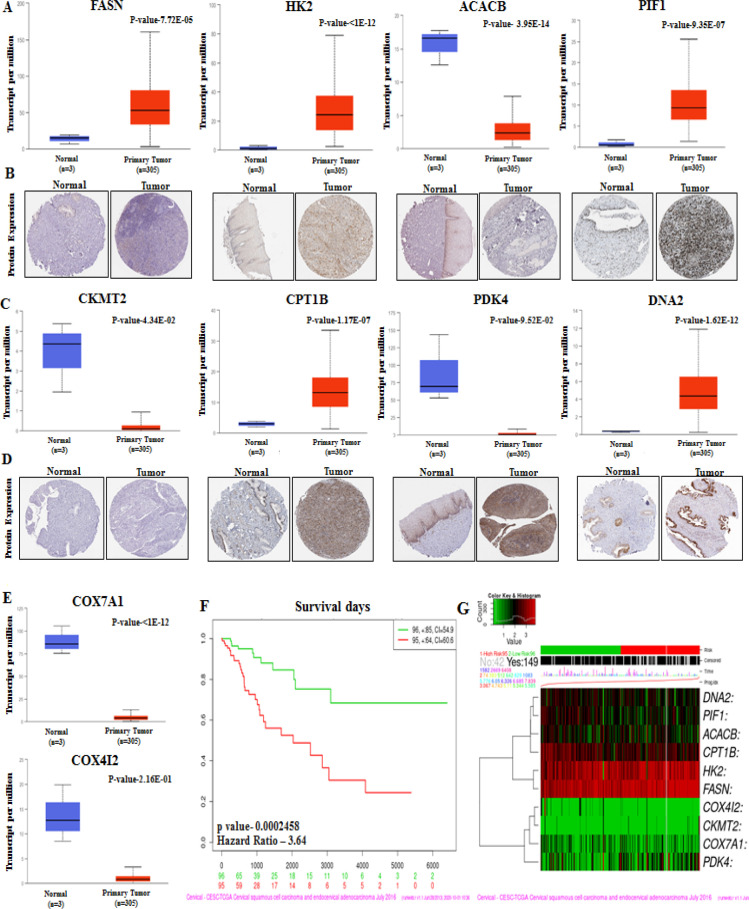
Validation of the HG Expression at Protein Level. A) The bar graph and IHC images represents the expression of HG at RNA and protein level. B) Survival analysis of hub genes performed by using SurvExpress. C) Heatmap showing the hub gene expression

The HG in the PPIN were predicted using CytoHubba and visualized using Cytoscape. The top 10 HG identified by CytoHubba includes FASN, HK2, ACACB, PIF1, COX7A1, CKMT2, CPT1B, PDK4, DNA2, and COX4I2. The HG analysis using SurvExpress showed that the low-risk group patients showed significantly higher survival than high-risk group patients (Risk group hazard ratio: 3.64, p=0.0002. Independent validation of HG expression was performed using Oncomine Research Edition (https://www.oncomine.org/resource/login.html) online tool (Rhodes et al., 2007). Oncomine analysis identified genes, namely, HK2, PIF1, COX7A1, CPT1B, DNA2, and COX4I2, were significantly differentially expressed in more than two independent datasets. Besides, 10 hub genes’ expression was also independently validated using the human protein atlas (HPA) database (https://www.proteinatlas.org/). Among the 10 genes, immunohistochemical data were available for only 8 genes (FASN, HK2, ACACB, PIF1, CKMT2, CPT1B, PDK4, and DNA2). The expression of the 8 genes was in concordance with TCGA-CESC expression data (Figure 7, Supplementary Table 5).

*Identification of Drugs targeting NEMG*

The potential druggable candidates in the 52 DE-NEMGs were predicted using the drug-gene interaction database (DGIdb; https://www.dgidb.org/). The DGI was evaluated for 52 DE-NEMG against approved, anti-neoplastic, and immunotherapeutic agents. Our analysis identified 195 drugs targeting 16 DE-NEMG (Supplementary Table 6). Drugs such as Cisplatin, Fluorouracil, Paclitaxel, Docetaxel, Vincristine, Adriamycin, and Doxorubicin are already being used to treat various cancers. Besides, our analysis also identified several novel drugs that can be repurposed to treat CC.

**Table 1:** NEMG and TCGA-CESC

Expression	Gene Name
Upregulated (27)	BDH1; SLC25A10; IDH2; DARS2; SCO2; COX6B2; GTP2; CPT1B; HK2; CKMT1A; TUBB3; FASN; TSTD1; RECQL4; DHRC24; NUDT5; NCEH1; PIF1; AGMAT; COMTD1; PMAIP1; TDRKH; CYP27B1; MGME1; CKMT1B; CDC25C; DNA2
Downregulated (25)	PDK4; COX7A1; MOCS1; ACSM1; CKMT2; COX4I2; DMGDH; ABCD2; ACACB; ACSS3; HSPB7; ABCA9; NT5DC3; MAOB; PPM1K; MSRB3; NIPSNAP3B; MGARP; DUSP26; EFHD1; BCL2; PABPC5; PRSS35; C10orf10; CLIC4
Methylation	
Hypermethylated (11)	CPT1B; DMGDH;CKMT2;ACSS3;COX7A1;MAOB;PDK4;PABPC5;MSRB3;MGARP;HSPB7
Hypomethylated genes (8)	CKMT1A; CKMT1B;TSTD1;AGMAT;COMTD1;SCO2;NCEH1;BCL2
Inverse correlation (Methylation vs Expression)	COX6B2;CYP27B1;CKMT1A;RECQL4;CKMT1B;PIF1;PMAIP1;TSTD1;AGMAT;GPT2;COMTD1;TDRKH;CPT1B;NT5DC3;MOCS1;DMGDH;COX4I2;CKMT2;PPM1K;ACSM1;ACSS3;COX7A1;PDK4;PABPC5;MSRB3;MGARP; HSPB7
Staging (16)	HK2;PIF1;DHCR24;SLC25A10;CPT1B;FASN;NCEH1;NIPSNAP3B;ABCD2;DMGDH;ACACB;DEPP1; ACSS3;MAOB;PDK4;PABPC5
Metastasis (10)	BDH1; CPT1B; CKMT1A; MSRB3; MGARP; PMAIP1;TDRKH;CKMT1B; PDK4; BCL2
Prognosis	
Methylation	OS: SCO2;MOCS1;DEPP1;ABCA9
	DFS: DEPP1
	PFS: NUDT5;DEPP1
Expression	OS: HK2;MSRB3;FASN; BDH1
	DFS: CKMT2

## Discussion

CC is one of the common gynecological problems in many developing and underdeveloped countries (Arbyn et al., 2020). Various risk factors such as infection with high-risk HPV, smoking, alcohol consumption, multiple sexual partners, parity, and oral contraceptives are well recognized to influence CC’s development and progression (Johnson et al., 2019; Wudtisan et al., 2019). Despite the availability of early diagnostic procedures and advances in CC treatment, there is little improvement in the disease prognosis. The detection of the disease at an advanced stage contributes to poor clinical outcomes and high mortality (Canfell et al., 2020). Besides, only limited prognostic markers are available for clinical use in CC. Hence, it is indispensable to explore new prognostic markers and additional target genes for CC treatment.

Mitochondria play a critical role in bioenergetics and cellular metabolism. Besides, mitochondria also participate in various signaling, programmed cell death, heme, and steroid synthesis (Shaughnessy et al., 2015). In humans, dysregulation of mitochondria’s structure and function is linked with all stages of cancer progression. Additionally, studies have reported the association between pathogenic mtDNA variations with CC (Sun et al., 2020). Moreover, mitochondrial defects can promote tumor growth and metastasis by providing energy to growing cancer cells, promoting cell survival via activation of anti-apoptotic signaling pathways. Both anterograde and retrograde signaling is critical for normal homeostasis of the cells. Defective NEMG signaling contributes and controls several aspects of carcinogenesis, such as growth, proliferation, migration, and metastasis. The abnormal expression of NEMG has been reported as a potential diagnostic and prognostic marker and a novel target for therapy in various cancers (Chatterjee et al., 2006; Indran et al., 2011). However, such studies are limited in CC. Hence, a comprehensive investigation of NEMG may significantly improve CC’s management by providing markers for diagnosis, prognosis, and treatment. Accordingly, in the present study, we identified the NEMG with potential prognostic applications in CC.

Cancer staging is vital for designing the treatment and predicting the prognosis. Towards this, using the UALCAN database, we predicted the potential of 52 DE-NEMG for CC staging. The 52 genes were significantly differentially expressed between normal and tumor samples, suggesting their potential in CC diagnosis. However, out of 52 DEGs, the differential expression of 16 genes could differentiate specific stages of CC. Thus, our study has identified a panel of genes whose expression profiling could be useful for staging CC and required to be evaluated further in clinical samples.

Metastasis is a critical factor for therapeutic resistance and poor patient survival. Thus, identifying the metastasis-associated genes is proposed to improve patient care. Towards this, we investigated the potential of 52 DE-NEMG and their contribution towards the induction of metastasis in CC. Among the 52 DE-NEMG, 10 genes have been linked to head and neck, lung, or lymph node metastasis in CC. Among the various metastatic genes identified, elevated expression of CPT1B is reported in chemoresistant metastatic breast tumors (Wang et al., 2018). An axis comprising of lncRNA n335586/miR-924/CKMT1A is reported in metastasis of hepatocellular carcinoma cells (Fan et al., 2018). Overexpression of MSRB3 is reported in peritoneal metastasis and poor prognosis in gastric cancer (Zhang et al., 2020). The role of PDK4 in colon cancer metastasis is also reported. The PDK4 mediated metabolic reprogramming contributes significantly to the metastasis cascade (Leclerc et al., 2017). BCL2 promotes and accelerates metastasis in breast and colorectal cancer (Um, 2016). In-silico analysis using HCMDB identified 10 proteins associated with CC metastasis based on clinical correlation analysis. Many of these genes are either directly or indirectly participating in metabolic reprogramming and/or adaptation to promote cancer cells’ growth and survival. However, more functional studies are required to identify the precise role of these genes in metastasis and metabolic reprogramming in CC. 

The role of abnormal DNA methylation in carcinogenesis is well established. Both promoter hypermethylation resulting in tumor suppressor gene silencing and hypomethylation activating oncogenes are reported to promote CC. Previous studies have indicated that measuring DNA methylation has the potential diagnostic and prognostic value in CC (Kabekkodu et al., 2014). Herein, we investigated the association between methylation and expression via correlation analysis. Our analysis identified 19 gene promoters were significantly differentially methylated between normal and tumor samples. Interestingly, 15 out of 19 NEMG showed an inverse correlation between methylation and expression. Moreover, our analysis recommends the use of SCO2, MOCS1, DEPP1, and ABCA9 for OS, and NUDT5, DEPP1 for PFS in CC after further validation.

A PPIN was constructed to better understand the interaction and functions of 52 DE-NEMG. PPIN identified 52 nodes and 13 edges, suggesting that these proteins’ interactions may have a critical role in CC development and progression. CytoHubba analysis identified FASN, HK2, ACACB, PIF1, COX7A1, CKMT2, CPT1B, PDK4, DNA2, and COX4I2 as critical members of the network or HG. Our study’s 10 HG were linked to fatty acid biosynthesis, AMPK signaling, insulin signaling, oxidative phosphorylation, and metabolic process. Abnormal activation or suppression of these pathways has already been linked to various cancers, including CC. The hub genes’ cross-validation using the Oncomine tool in 5 independent data sets identified HK2, PIF1, COX7A1, CPT1B, DNA2, and COX4I2 as commonly altered in CC. Thus, abnormal expression of these genes might be critical for CC pathogenesis. The details of these hub genes commonly identified in multiple datasets are described below.

The conversion of glucose to glucose-6-phosphate is mediated by HK2 (Hexokinase 2) enzyme. Cancer cells activate glycolytic enzymes to promote metabolic changes to support the growth and proliferation of cancer cells. HK2 is often upregulated in numerous cancers, including CC. HK2 overexpression promotes proliferation, migration, and inhibition of apoptosis in SiHa cells via phosphorylation of AKT. High HK2 expression correlates with the size of the tumor, pathological grade, and prognosis (Liu et al., 2019). Inhibition of HK2 sensitized the CC cells to radiation and induced apoptosis via caspase-3 and PARP cleavage activation. Thus, targeting HK2 can be used to sensitize cancer cells to radiotherapy and control tumor cells’ growth and proliferation (Liu et al., 2017). PIF1 (5’-To-3’ DNA Helicase) encodes for a protein with 5’ to 3’ DNA helicase function required to maintain nuclear and mtDNA genome stability. PIF1 acts as a tumor promoter in CC via suppressing the TERT. CC cells show a high expression of PIF1 and promote proliferation by inhibiting apoptosis by targeting BAX and Caspase-3 (Wang et al., 2020).

COX7A1 (Cytochrome C Oxidase Subunit 7A1) takes part in a biochemical reaction involving electron transfer activities during the OXPHOS reaction. COX7A1 acts as a tumor suppressor by inhibiting non-small cell lung cancer’s growth and proliferation by induction of apoptosis via blocking autophagic flux (Zhao et al., 2019). Another study demonstrated the downregulation of COX7A1 by promoter hypermethylation in breast cancer (He et al., 2019). Besides, COX7A1 is implicated in tumor metabolism and therapy. However, their precise role in CC is yet to be established. CPT1B (Carnitine Palmitoyltransferase 1B) is a rate-controlling enzyme that participates in the transfer of long-chain fatty acyl-CoAs from the cytoplasm to mitochondria. Yeh and colleagues in 2006 demonstrated the overexpression of this gene in colorectal cancer (Yeh et al., 2006). In muscle-invasive bladder cancer, CPT1B deregulation correlated with a higher rate of mortality (Kim et al., 2016). In our study, we found overexpression of CPT1B in CC. This suggests that the overexpression of CPT1B leading to metabolic alterations may be a prerequisite for cancer cells’ growth and survival. In gastrointestinal cancer, higher expression of CPT1B displayed a superior response to Carnitine palmitoyltransferase inhibitors (Wang et al., 2020). Furthermore, targeting CPT1B in cancers with abnormal lipid metabolism and fatty acid oxidation (castration-resistant prostate cancer) is proposed. CPT1B overexpression is correlated with poor prognosis in prostate cancer (Abudurexiti et al., 2020). However, the role of CPT1B in CC is yet to be uncovered.

DNA2 (DNA Replication Helicase/Nuclease 2) is a protein required to maintain mitochondrial and nuclear DNA stability by participating in the replication and repair process. Similar to our findings, Li et al. 2018 reported the upregulation of DNA2 in CC (Li et al., 2018). However, the molecular mechanism and pathways regulated by DNA2 in CC are still elusive. Overexpression of DNA2 is reported in multiple human cancers. By counteracting the replication stress, DNA2 can act as a cancer promoter (Zheng et al., 2020). Targeting DNA2 is proposed as a therapeutic target to control tumor growth in pancreatic cancer (Kumar et al., 2017). Thus, DNA2 may act as a potential driver of carcinogenesis. COX4I2 (Cytochrome C Oxidase Subunit 4I2) is an enzyme that drives oxidative phosphorylation and is downregulated in our analysis. Hypoxic condition induces COX4I2 expression as it harbors hypoxia-responsive elements. In HeLa cells, hypoxia enhances the promoter activity of COX4I2 (Fukuda et al., 2007).

Therapy resistance is one of the major problems in cancer treatment. To overcome the drug resistance, researchers are looking for new targets and repurposing of the existing drugs. Herein, we have tested the potential druggable DE-NEMG using the DGIdb database. Our study leads to the identification of 16 druggable genes and 195 drugs. Based on our analysis, we propose the use of a combination of drugs for treating CC. The various known drugs identified in our study, which are already in use for treating cancer in general and CC in particular, include Cisplatin, Fluorouracil, Paclitaxel, Docetaxel, Vincristine, Adriamycin, and Doxorubicin.

The present study has some limitations. CC has several histological types. The data used in the study included all histological types without performing any subgroup analysis. Besides, our survival analysis also contained data for all histological types of CC without performing subgroup analysis. Lack of experimental validation is another limitation of our study.

Nevertheless, our study is the first comprehensive study investigating the role of DE-NEMG as potential markers for prognostic application in CC. Our in silico investigation identified new insights into the interaction of NEMG and associated pathways regulated during CC. Although we have analyzed many tumor samples, the normal samples included are still inadequate, suggesting the need for further experimental validation before further conclusions are drawn. Besides, several genes reported as significantly differentially expressed in TCGA-CESC data require further validation in patient cohort and functional studies using in vitro and in vivo models.

In the present study, we assessed transcriptomic and corresponding clinical data from the TCGA-CESC dataset and identified 52 significantly DE-NEMGs. The prognostic utility, functional and pathway enrichment, and PPIN were performed using in silico methods. The PPIN constructed suggested the novel PPIN in CC. The findings of our study have potential prognostic implications in CC. Our DGI analysis identified novel drugs that can be repurposed to treat CC. The druggable genes identified in our study may facilitate the development of specific and more effective treatments against CC.

## Author Contribution Statement

SPK designed the study; CM and SE acquired, analyzed, and interpreted the data; DA, NKG and SS drafted the initial manuscript. SM, SC and SPK helped in the critical revisions of the manuscript. All the authors have read and approved the final draft of the manuscript.
